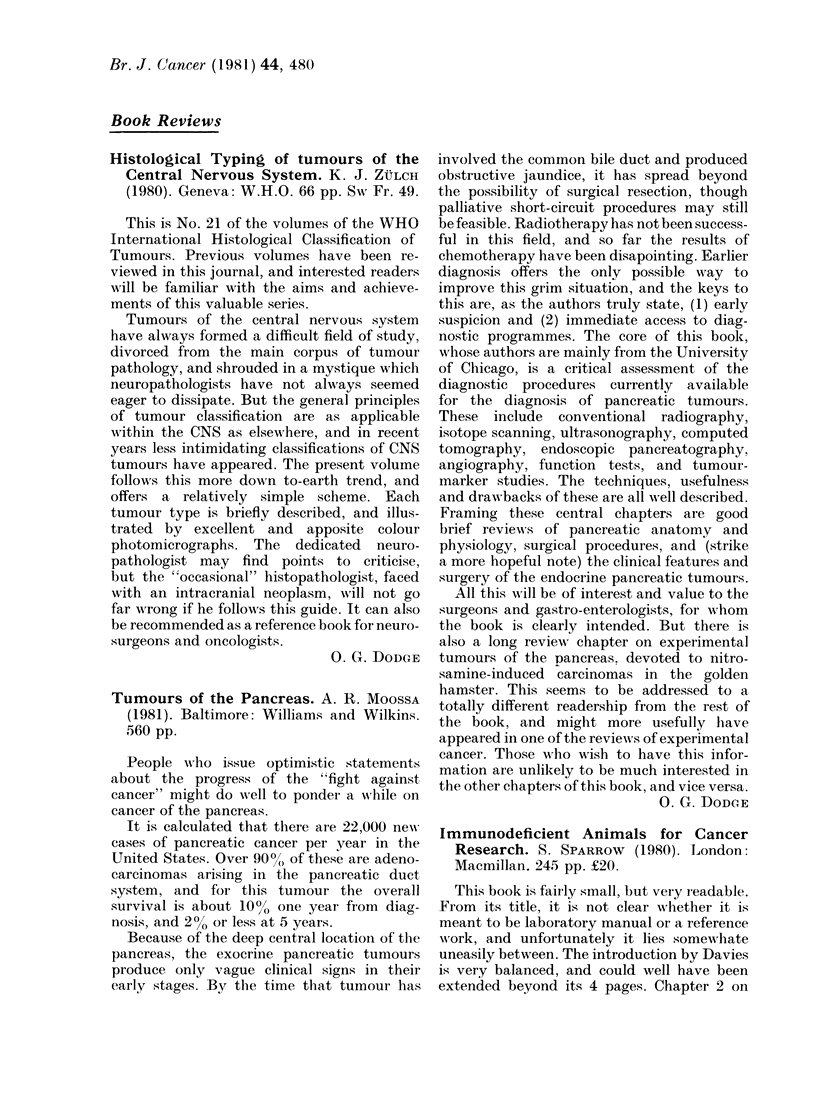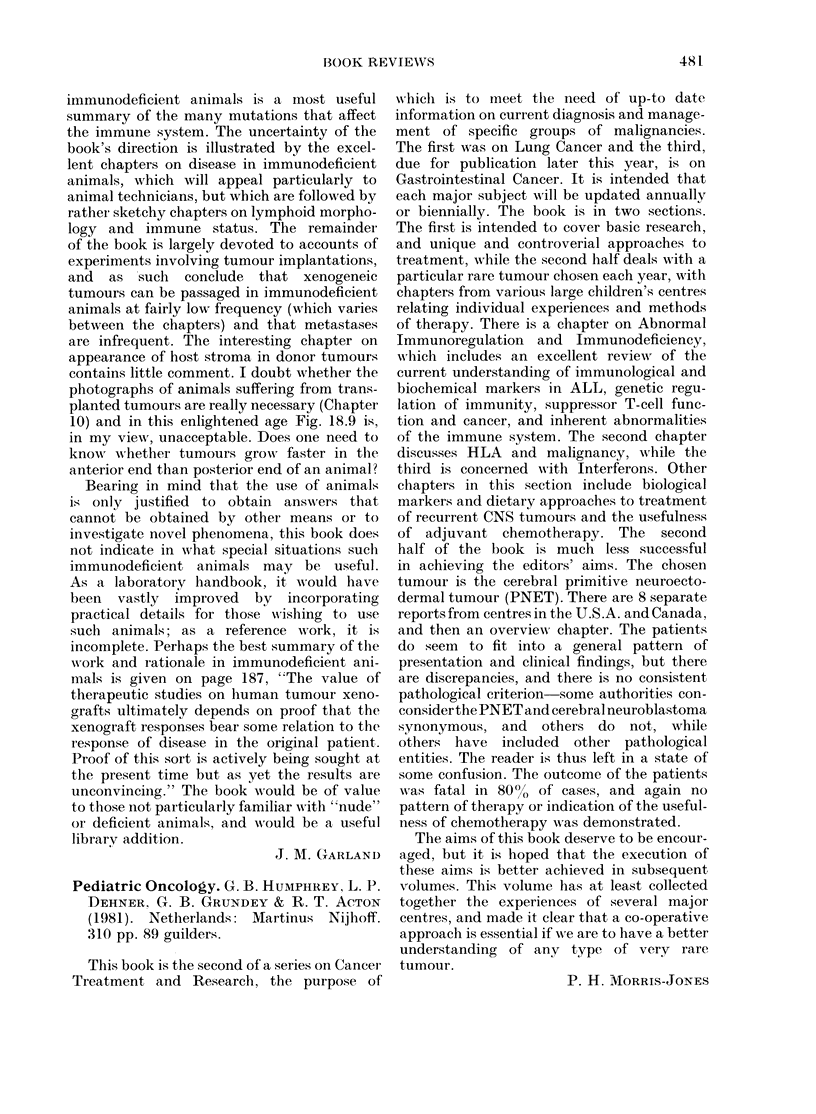# Immunodeficient Animals for Cancer Research

**Published:** 1981-09

**Authors:** J. M. Garland


**Immunodeficient Animals for Cancer Research.** S. Sparrow (1980). London: Macmillan. 245 pp. £20.

This book is fairly small, but very readable. From its title, it is not clear whether it is meant to be laboratory manual or a reference work, and unfortunately it lies somewhate uneasily between. The introduction by Davies is very balanced, and could well have been extended beyond its 4 pages. Chapter 2 on immunodeficient animals is a most useful summary of the many mutations that affect the immune system. The uncertainty of the book's direction is illustrated by the excellent chapters on disease in immunodeficient animals, which will appeal particularly to animal technicians, but which are followed by rather sketchy chapters on lymphoid morphology and immune status. The remainder of the book is largely devoted to accounts of experiments involving tumour implantations, and as such conclude that xenogeneic tumours can be passaged in immunodeficient animals at fairly low frequency (which varies between the chapters) and that metastases are infrequent. The interesting chapter on appearance of host stroma in donor tumours contains little comment. I doubt whether the photographs of animals suffering from transplanted tumours are really necessary (Chapter 10) and in this enlightened age Fig. 18.9 is, in my view, unacceptable. Does one need to know whether tumours grow faster in the anterior end than posterior end of an animal?

Bearing in mind that the use of animals is only justified to obtain answers that cannot be obtained by other means or to investigate novel phenomena, this book does not indicate in what special situations such immunodeficient animals may be useful. As a laboratory handbook, it would have been vastly improved by incorporating practical details for those wishing to use such animals; as a reference work, it is incomplete. Perhaps the best summary of the work and rationale in immunodeficient animals is given on page 187, "The value of therapeutic studies on human tumour xenografts ultimately depends on proof that the xenograft responses bear some relation to the response of disease in the original patient. Proof of this sort is actively being sought at the present time but as yet the results are unconvincing." The book would be of value to those not particularly familiar with "nude" or deficient animals, and would be a useful library addition.

J. M. GARLAND